# Exploring stress-tolerant plant growth-promoting rhizobacteria from groundnut rhizosphere soil in semi-arid regions of Ethiopia

**DOI:** 10.1080/15592324.2024.2365574

**Published:** 2024-06-24

**Authors:** Asnake Beshah, Driba Muleta, Gudina Legese, Fassil Assefa

**Affiliations:** a Department of Cellular, Microbial and Molecular Biology, Addis Ababa University, Addis Ababa, Ethiopia; b Biotechnology Institute, Addis Ababa University, Addis Ababa, Ethiopia; c Center for Environmental Science, Addis Ababa University, Addis Ababa, Ethiopia

**Keywords:** Bio-inoculants, drought stress, PGPR, phosphate solubilization, rhizobacteria

## Abstract

The potential of rhizobacteria with plant growth promoting (PGP) traits in alleviating abiotic stresses, especially drought, is significant. However, their exploitation in the semi-arid regions of Ethiopian soils remains largely unexplored. This research aimed to isolate and evaluate the PGP potential of bacterial isolates collected from groundnut cultivation areas in Ethiopia. Multiple traits were assessed, including phosphate solubilization, indole-3-acetic acid (IAA) production, ammonia production, salt and heavy metal tolerance, drought tolerance, enzyme activities, hydrogen cyanide production, antibiotic resistance, and antagonistic activity against fungal pathogens. The identification of potent isolates was carried out using MALDI-TOF MS. Out of the 82 isolates, 63 were gram-negative and 19 were gram-positive. Among them, 19 isolates exhibited phosphate solubilization, with AAURB 34 demonstrating the highest efficiency, followed by AURB 12. Fifty-six isolates produce IAA in varying amounts and all isolates produce ammonia with AAURB12, AAURB19, and AAURB34 displaying strong production. Most isolates demonstrated tolerance to temperatures up to 40°C and salt concentrations up to 3%. Notably, AAURB12 and AAURB34 exhibited remarkable drought tolerance at an osmotic potential of −2.70 Mpa. When subjected to levels above 40%, the tested isolates moderately produced lytic enzymes and hydrogen cyanide. The isolates displayed resistance to antibiotics, except gentamicin, and all isolates demonstrated resistance to zinc, with 81–91% showing resistance to other heavy metals. AAURB34 and AAURB12 exhibited suppression against fungal pathogens, with percent inhibition of 38% and 46%, respectively. Using MALDI-TOF MS, the promising PGP isolates were identified as Bacillus megaterium, Bacillus pumilus, and Enterobacter asburiae. This study provides valuable insights into the potential of rhizobacteria as PGP agents for mitigating abiotic stresses and contribute to the understanding of sustainable agricultural practices in Ethiopia and similar regions facing comparable challenges.

## 1. Introduction

The rhizosphere, the region surrounding plant roots, harbors a complex microbial community. These beneficial microorganisms, residing in the rhizosphere, establish symbiotic relationships with plants and contribute to their overall health and resilience.^1^ Among these microorganisms, Plant Growth Promoting Rhizobacteria (PGPR) have gained significant attention due to their ability to enhance plant growth and alleviate various environmental stresses. In agricultural systems, harnessing the potential of PGPR has gained significant attention as an eco-friendly approach to sustainable crop production.^2^

Ethiopia, with its diverse agro-ecological zones and agricultural practices, offers a rich source of microbial biodiversity in crops rhizosphere including groundnut.^3^ Groundnut (*Arachis hypogaea*) is one of the major oilseed crops cultivated in the country, providing a valuable source of nutrition and income for farmers.^4^ However, groundnut production is frequently challenged by various abiotic stresses, such as drought, salinity, and nutrient deficiencies, which can limit crop yield and quality.^5^

The rhizosphere of groundnut plants represents a unique niche that harbors a diverse microbial community, including PGPR with the potential to enhance plant growth and tolerance to diverse stresses.^6^ Understanding and harnessing the capabilities of stress-tolerant PGPR from the groundnut rhizosphere in Ethiopia can provide valuable insights for developing sustainable agricultural practices and improving groundnut production in some semi-arid parts of the country.

In recent years, there has been a growing interest in isolating and characterizing stress-tolerant PGPR strains from the groundnut rhizosphere. These PGPR strains possess unique traits that enable them to thrive in the challenging soil environment to provide beneficial effects to the host plant. Understanding the diversity and functional attributes of these stress-tolerant PGPR strains can lead to the development of sustainable agricultural practices and the improvement of groundnut crop productivity.

The objective of this study was to isolate, characterize, and identify stress-tolerant PGPR strains from the rhizosphere of groundnut plants in Ethiopia. The isolated strains were evaluated for their ability to promote plant growth, tolerate abiotic stresses, and exhibit beneficial traits such as nitrogen fixation, phosphate solubilization, and production of plant growth-promoting substances.

The findings of this study have significant implications for sustainable agriculture in Ethiopia and beyond. The identification of stress-tolerant PGPR strains from the groundnut rhizosphere can serve as a valuable resource for developing bio inoculants and eco-friendly agricultural practices to enhance groundnut productivity and to mitigate the adverse effects of abiotic stresses. Furthermore, understanding the diversity and functional traits of these PGPR strains can contribute to the broader field of plant–microbe interactions and provide insights into the mechanisms underlying plant growth promotion and stress tolerance.

## 2. Materials and methods

### 2.1. Isolation of PGP bacteria

Sixty geo-referenced soil samples were randomly collected at a depth of 20 cm from groundnut fields with different agro-ecologies area of Oromia, Benishangul-Gumuz and Amhara National Regional States of Ethiopia. The collection sites are located from 1069 to 1640 m above sea level with pH range of 5.2 to 6.9 and the soil samples collected were characterized as sandy loam. The soils were used to trap plant growth promoting bacteria using groundnut variety,” Babile1. Healthy, undamaged, and surface sterilized by using ethanol and hydrogen peroxide seed were planted in side a pot having a capacity to contain 3 kg of soil under greenhouse conditions.

For isolation of rhizosphere bacteria, 1.0 g of the soil was taken and mixed in 9.0 ml of sterile distilled water and vortexed for 5 min.^7^ For isolation of root adhering (rhizoplane) bacteria, fresh roots were washed using distilled water. Soil suspension and root washed solutions were serially diluted. A 100 μl of supernatant from each dilution of rhizosphere soil and root washing solutions were transferred onto nutrient agar medium and incubated at optimum temperature. The purified bacteria cultures were identified based on their morphology (colonial and cellular) following Berge’s Manual of Determinative Bacteriology^8^ and screened for different PGP traits, biocontrol properties, and abiotic stress tolerance activities.^9^

### 2.2. In vitro screening for plant growth promoting traits

#### 2.2.1. Phosphate solubilization

The phosphate solubilizing activity of all the PGPR isolates was assessed *in vitro* using Pikovaskaya’s medium. Spot-inoculation of the cultures was performed on Pikovskaya’s medium plates followed by incubation at 30°C for 7 days. The presence of a clear zone surrounding bacterial growth indicated a positive outcome for phosphate solubilization test.^10^

To determine the ability of bacteria to solubilize TCP (tricalcium phosphate), the solubilization halo diameter was measured around the colonies after inoculating a fresh bacterial suspension on PVK agar medium. The solubilization index (SI) was calculated after 7, 14, and 21 days of incubation at 28 ± 2°C using the formula of.^11^

**SI = (CD + HD)/CD,**

Where CD represents the colony diameter and HD represents the halo zone diameter.

The quantification of phosphate release was carried out utilizing the chlorostannous reduced molybdophosphoric acid blue method, as outlined by.^12^ In brief, a 1 mL aliquot of the bacterial culture was introduced into a 100 mL sterile Pikovskaya broth, contained in an Erlenmeyer flask. The flask was then incubated at a temperature of 28 ± 2°C for a duration of 11 days, with continuous shaking at a rate of 120 rpm. To serve as a control, an uninoculated broth was also prepared. The entire experimental procedure was conducted in triplicates to ensure accuracy and reliability.

At specific time intervals (3rd, 5th, 7th, and 10th day), 10 mL of broth was extracted from each sample to measure the soluble phosphorus and pH variation. The extracted cultures were centrifuged at a speed of 10,000 rpm for 15 min. Following centrifugation, 100 µL of the supernatant was added to a flask containing 10 mL of chloromolybdic reagent, while maintaining a shaking condition. The mixture was subsequently diluted with 40 mL of distilled water. To complete the process, 5 drops of chlorostannous acid reagent were carefully added along the sides of the flask and thoroughly mixed. The final volume was adjusted to 50 mL using distilled water.

The resulting blue coloration was measured spectrophotometrically at a wavelength of 660 nm, with a blank used as a reference. A standard curve was established within the range of 10–50 µg/mL to accurately determine the concentration of phosphate in the samples.

#### 2.2.2. IAA production

Rhizobacterial cultures were grown on 100 ml Luria Bertani (LB) broth amended with 1 mg/l tryptophan as the precursor of IAA and incubated in a shaker at 120 rpm at 30°C for 3–5 days. Each rhizobacterial strain was inoculated in triplicate. Fully grown cultures were centrifuged at 10,000 rpm for 10 min. A 2 ml of the supernatant was mixed with 4 ml of the salkowski reagent (50 ml, 35% of HClO4 (perchloric acid), 1 ml 0.5 m FeCl3 solution). Appearances of pink color in test tube were an indicator of positive result for IAA production.^13^

#### 2.2.3. Ammonia production

All the isolates were tested for ammonia production by inoculating the cultures in 10 ml peptone water and incubated for 72 h at 36°C. After incubation, a 0.5 ml of Nessler’s reagent (by dissolving 100 gm mercuric iodide and 70 gm of potassium iodide in a small amount of water. By adding this mixture slowly, with stirring, to a cooled solution of 160 gm NaOH in 500 ml of water and dilute the mixture in 1 liter was added to each tube. Development of brown to yellow color was taken as a positive test for production of ammonia^14^

### 2.3. In vitro screening for stress tolerance

#### 2.3.1. Temperature

The growth of each isolate at different incubation temperatures was evaluated by inoculating each isolate onto nutrient agar plates. The inoculated plates were incubated at a temperature of 4°C, 10°C, 15°C, 20°C, 25°C, 30°C, 35°C, 40°C and 45°C and as indicated in.^15^

#### 2.3.2. Salt

To assess the growth capabilities of the isolates under varying salt concentrations, the following methodology was employed: Each isolate was inoculated onto a nutrient agar medium supplemented with different NaCl concentrations, namely 1%, 4%, 5%, 6%, 7%, 8%, 9%, and 10%. As a control, a concentration of 0.1% NaCl was utilized, as specified in the study by.^16^ The growth of the isolates was subsequently evaluated based on their performance in these different salt environments.

#### 2.3.3. Heavy metals

To examine the response of all the isolates, they were subjected to a solid nutrient agar medium supplemented with various heavy metals that had been sterilized through a 0.2 μm pore size membrane. The concentrations of the respective heavy metals in μg ml-1 were as follows: AlK2SO4 (250), CoCl2 (20), CuCl2 (50), FeCl3.6H2O (750), MnSO4 (500), and ZnCl2 (50). The pH of the medium was maintained at 6.8, as indicated.^17^ The PGPR culture suspensions of 72-h old was streaked on the media (plates) and the growth of colonies was recorded as (+) for growth and (-) for no growth.

#### 2.3.4. Drought

To characterize drought tolerance of the isolates, 10 mL of trypticase soya broth amended with 0%, 10%, 20%, 30% and 40% of polyethylene glycol-6000 to have different water potentials −0.05, −0.15, −0.30, −0.49, and −0.73 MPa, respectively.^18^ For each isolate, 0.1 mL of freshly grown cultures of the test strains was inoculated into each broth. The inoculated tubes were incubated at 28 ± 2°C for 24 h, and OD was recorded after 3 days. OD values of drought tolerance were determined as follows: completely sensitive OD < 0.3; sensitive OD= (0.3–0.39); tolerant OD= (0.4–0.5), and completely tolerant OD > 0.5.^19^

### 2.4. In vitro screening for bio-control traits

#### 2.4.1. Hydrolytic enzyme production

##### 2.4.1.1. Protease production test

The bacterial isolates were screened for their ability to produce proteolytic enzymes onto skim milk agar (SMA) medium.^20^ Clear zone formation around the bacterial colonies after 48 h of incubation at 30°C was considered as a positive result and the diameter of the clear zone was also measured.

##### 2.4.1.2. Cellulase production test

Spot inoculation of the PGPR broth culture was made on the cellulose agar medium containing KH_2_PO_4_ 0.5 g, MgSO_4_ 0.25 g, cellulose 2 g, agar 15 g, and gelatin 2 g; distilled water 1 l and at pH 6.8–7.2 Formation of clear halo zone on cellulose medium was a positive indicator for cellulase synthesis.^21^

##### 2.4.1.3. HCN production

Rhizobacterial isolates were screened for HCN production. Each isolate was inoculated onto the nutrient medium containing 4.4 g glycine per liter. To the top of the plate, Whatman filter paper no. 1 soaked in 2% sodium carbonate in 0.5% picric acid solution was placed and sealed with parafilm. The plates were incubated at 30°C for 4 days and color change of the filter paper from deep yellow to reddish-brown was a good indicator for HCN production.^22^

#### 2.4.2. Intrinsic antibiotic resistance (IAR)

Resistance of isolates to different antibiotics at different concentrations was evaluated. The antibiotics used were Tetracycline, Erythromycin, Ampicillin, Chloramphenicol, Gentamicin and Penicillin. Each antibiotic was tested at the following concentrations in μg/ml (2.5, 5 and 10). Erythromycin was dissolved in ethanol, whereas others were dissolved in sterilized distilled water. The stock solution of each antibiotic was prepared by dissolving 2 g of each antibiotic in 100 ml of water according to.^23^ The required concentration was aseptically added to the medium using different pipettes for each antibiotic. The sterilized solution of each antibiotic was added to autoclaved nutrient medium aseptically and shaken well. Then, the isolates were streaked on the plates prepared with antibiotics and incubate at 28 ± 2°C for 3–5 days and growth was checked during the incubation period.

#### 2.4.3. Antifungal activity

The antifungal activity of all the PGPR isolates were tested using a dual culture method^24^ A 10 µL of each PGPR isolate (10^6^ cells mL^−1^) was spot-inoculated onto the surface of potato dextrose agar (PDA)-nutrient agar (1:1[w/w]) petri dishes (90 mm diameter) at a distance of 3 cm from the center and at four equidistant points (12, 3, 9 and 6 o’clock positions). The plate was incubated at 28°C for 48 h. The test pathogen *Fusarium oxysporum* was obtained from Ethiopian Institute of Agricultural Research (EIAR) and placed at the center of each plate (including rhizobacterial-free control plates). Plates incubated under the same conditions until the fungus grew to the outer-edge of the control plates. Percentage inhibition of radial growth (PIRG) was calculated as [(C-T)/C] ×100, where: C is radial growth (mm) of fungus on control plates and T is the radial growth of the fungus in the dual culture.^25^

### 2.5. Identification of bacteria using MALDI-TOF

In order to identify rhizobacterial isolates using the MALDI-TOF MS method, a sterile toothpick was used to pick a single pure colony from the nutrient agar of each isolate. The colony was then placed onto a specialized steel 96 micro scout plate (MSP) (Bruker Daltonics) using the direct transfer method. A thin film of the colony was spread onto the wells of the plate. Afterwards, the plate was allowed to dry and a solution of α-cyano-4-hydroxycinnamic acid (1 μl CHCA) matrix was added. This matrix solution contained 12.5 mg/ml CHCA in a mixture of 50% acetonitrile (CAN) and 2.5% trifluoroacetic acid (TFA). The plate was left to dry completely at room temperature. The MALDI 96 MSP was then placed into the MALDI-TOF MS device, and the system was operated using the optimized method for identifying microorganisms. The device was set to the linear positive ion mode within a mass range of 2,000–20,000 Dalton (Da). A 60 Hz nitrogen laser with a wavelength of 337 nm was used as the ion source. Laser pulses consisting of 40 packets of 240 were applied to measure each colony.^26^ To ensure accuracy, each sample was studied in triplicate, providing multiple spectra for analysis.

### 2.6. Statistical analysis

All tests were performed with three replicates for each treatment. The data were tested at least twice with the same results. Treatment groups were evaluated using ANOVA and the test for least significant differences at a probability threshold of 5% (*p* < 0:05).

## 3. Results

### 3.1. Isolation of PGP bacteria

**Figure 1.**
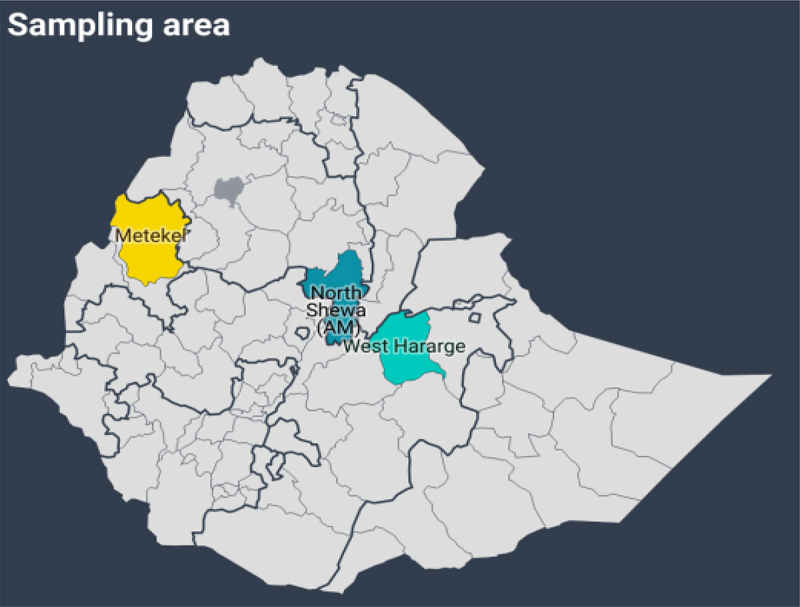
Geographical location of groundnut rhizosphere soil collection sites.

In this study, eighty-two (82) bacterial isolates were collected from groundnut growing soil of Ethiopia (Figure 1). Among these sixty-three isolates were gram negative and nineteen isolates were gram positive. All these isolates were screened for their plant growth promoting traits, stress tolerance ability and bio-control traits.

### 3.2. In vitro screening for plant growth promoting traits

#### 3.2.1. Phosphate solubilization

**Figure 2.**
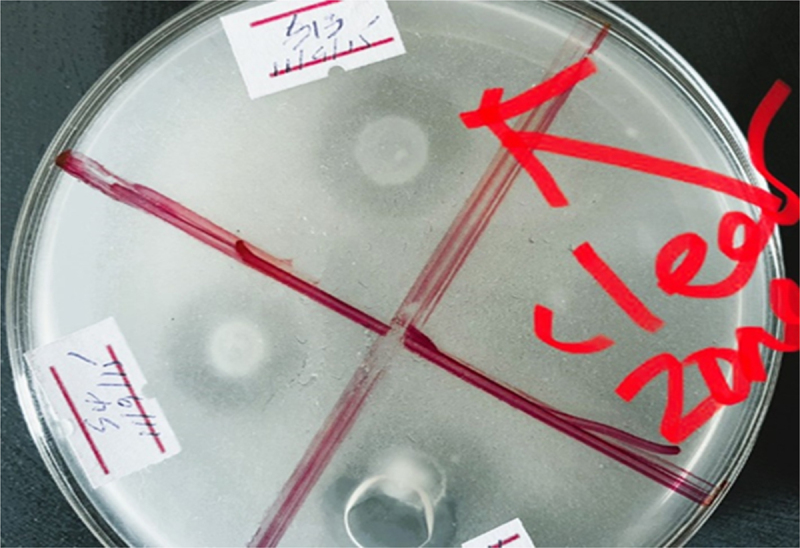
Growth of bacterial isolates on Pikovskaya medium. Bacterial isolates exhibit the formation of a clear zone around their colonies, indicating the ability to degrade the components present in Pikovskaya media.

A total of 19 isolates were found to solubilize Ca _3_(PO4)_2_ in pikovskaya (PVK) agar medium with various SI that ranged from 1.75 to 2.7. The highest SI activity (*p* < 0.01) exhibited by the isolates AAURB 34 with the value of 2.7 followed by AAURB 12 (2.5) and AAURB 11 (2.4). Among the 19 phosphate solubilizing isolates, AAURB 02 showed the least SI activity with the value 1.75 (Figure 2).

The statistical analysis revealed a significant difference in the means of the SI values obtained for the PVK media (*p* < 0.01), indicating a notable variation among the results. Additionally, the SI values of the same isolates at different pH levels were also found to be significantly different (*p* < 0.01). These findings highlight the importance of limiting the use of the halo method as a preliminary screening tool for PSB. As a result, the isolated and purified bacteria were further assessed in a liquid medium, with a specific focus on quantifying the release of soluble phosphorus

The findings of the study revealed that among the 82 isolates examined, only 19 isolates exhibited the ability to solubilize phosphorus from an insoluble phosphate source present in the media with varying amounts. The utilization of the colorimetric method allowed for the differentiation between phosphate solubilizing isolates, which displayed a blue color, and the control group, which appeared yellow upon the addition of chlorostannous reagent on the 5th day of the assay. Notably, the isolate labeled as AURB-34 exhibited the highest solubilization concentration during the assessment.

**Figure 3.**
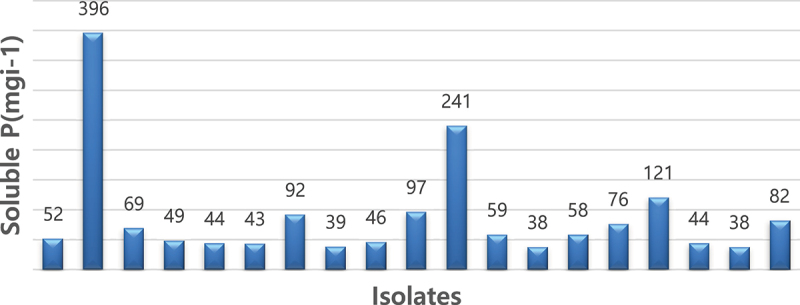
Quantitative estimation of selected PGPR isolates for P solubilization in liquid medium.

These isolates demonstrated the potential to solubilize phosphate from Pikovskaya’s media within a range of concentrations. Specifically, on the 3rd and 5th days, phosphate solubilization ranged from 37 to 264.4 µg/mL, while on the 7th day, isolate AURB-34 (*Bacillus pumilus*) achieved a notable phosphate solubilization concentration of 396.48 μg/mL followed by AURB12 (*Bacillus megatrium*) with a phosphate solubilized amount of 240.92 μg/ml (Figure 3). As the experiment progressed beyond the 7th day, the amount of free phosphate gradually diminished during the phosphate solubilization process executed by the isolates. In order to accurately quantify the solubilized phosphate, a standard curve was constructed using tri-calcium phosphate (TCP) within the range of 50–500 μg/mL.

The analysis of the data revealed that all values obtained in the PVK culture medium were significantly different from the initial pH (*p* < 0.01, one-way ANOVA). Furthermore, there was a significant difference in the means of the pH values at three and seven days (*p* < 0.01), suggesting a time-dependent effect. However, there was no significant difference observed among the different species at both three- and seven-day time points (*p* = 0.16 and 0.18, respectively, as determined by the one-way ANOVA).

#### 3.2.2. IAA production

**Figure 4.**
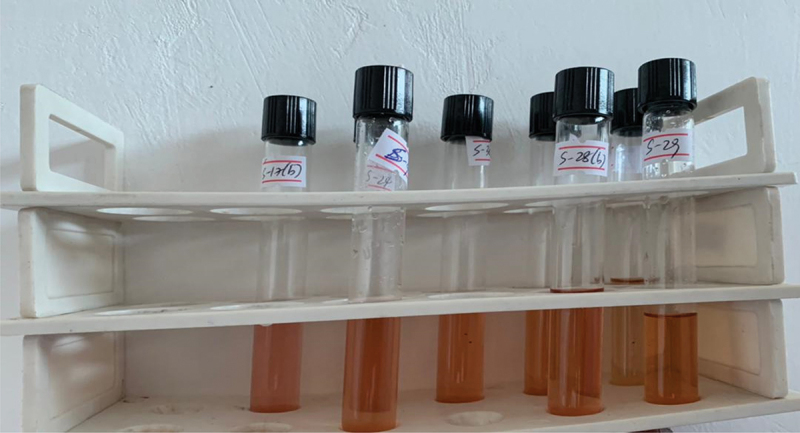
Detection of Indole-3-acetic acid (IAA) production in non-fluorescent bacteria (NFB) using Salkowski’s reagent.

In terms of IAA production, out of all the tested isolates, it was found that 56 PGPR isolates were able to produce IAA (Figure 4). Among the isolates, eight showed a deep pink color, while 25 and 23 isolates showed moderate and light pink colors, respectively.

The PGPR strains exhibited different intensities of pink color with varied amounts of IAA that ranged from 5.5 to 84.6 μg ml^−1^. Isolates AAURB28 and AAURB34 were identified as the highest IAA producers, while isolates AAURB40, AAURB51, and AAURB61 were found to be the least IAA producers, with values of 5.6, 5.5, and 5.5 μg ml-1, respectively (Table 1).

**Table 1.** Quantitative estimation of bacterial isolates for P solubilization and IAA production.

PGPR isolates	Soluble P (mg l−1)	Solublization index %	IAA Produced μg.ml−1
AURB10	82.14 + 5.6	2.3	43.6 + 4.3
AURB11	96.57 ± 0.56	2.4	60.3 + 3.1
AURB12	240.92 ± 2.4	2.5	77.3 + 0.62
AURB14	43.55 + 0.11	2	21.2 ± 0.77
AURB15	48.78 ± 4.6	2	32.4 + 23.1
AURB19	92.13± 6.6	2.25	67.4 + 10.6
AURB2	37.74 ± 3.9	1.75	34.8 + 0.66
AURB20	44.14 ± 4.95	2.3	45.6+ 32.1
AURB25	46.14 + 0.99	2	66.1+ 0.52
AURB27	76.42 + 0.6	2.5	32.6+ 0.11
AURB28	120.73 ± 0.7	2.6	84.6+ 2.7
AURB29	69.1 ± 8.3	2.3	30.7+ 5.77
AURB31	38.55 + 6.68	2.3	36.9 ± 0.81
AURB34	396.48 ± 7.2	2.7	82.3 ± 1.8
AURB35	37 ± 3.1	2	44.4 ± 11.5
AURB39	44.07 ± 7.8	2.25	36.4+ 0.63
AURB43	58.47 ± 2.7	2.3	32.4+ 0.33
AURB54	58.73 ± 8.6	2	49.2 ± 5.3
AURB6	51.82 + 2.9	2	54.6 ± 0.71

#### 3.2.3. Ammonia production

All the tested isolates changed the color of the medium to brown or yellow following the addition of Nessler’s reagents. Even if all the strains produced ammonia, they were differing in intensity and grouped as +++ (strong), ++ (moderate) and + (weak). Owing to this, isolates AAURB 12, AAURB 19 and AAURB 34 were categorized as a strong ammonia producer but isolates AAURB6, AAURB20, AAURB43 produced moderately, and the rest isolates categorized as weak producers.

### 3.3. In vitro screening for stress tolerance

#### 3.3.1. Temperature

**Figure 5.**
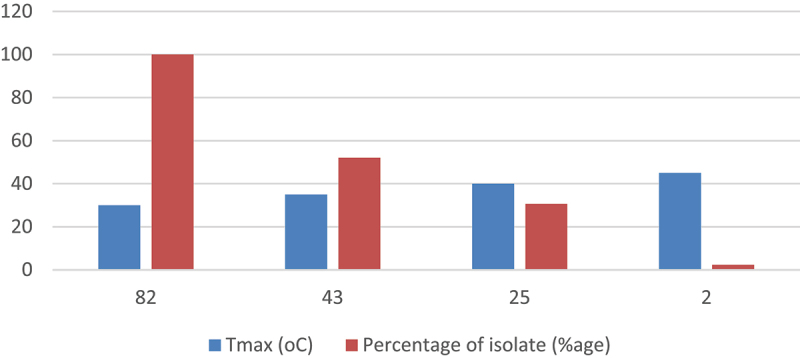
Temperature tolerance of tested PGPR isolates.

All the isolates were able to grow well at 30°C. As indicated in (Figure 5) almost half of the isolates were tolerant to 35°C, and 30% of the isolates were tolerant to temperatures of 40°C, but only two isolates GNR-12 and GNR-34 were able to grow at 45°C

#### 3.3.2. Salt

**Figure 6.**
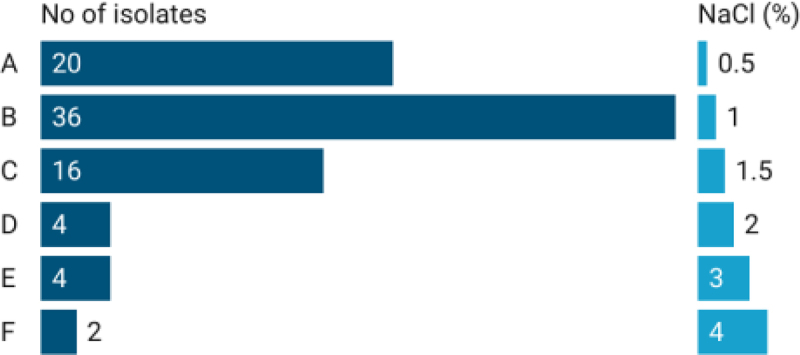
Salt tolerance of tested PGPR isolates.

All the tested rhizo-bacterial isolates were able to grow at 0.5% NaCl concentration. As the concentration of NaCl increased, the growth of the isolates was found to be inhibited. Two bacterial isolates AAUR 54 and AAUR 31 were found to be tolerant to a salt concentration of 4% followed by AAUR 12, AAUR3 4 and AAUR 37 with 3% concentration (Figure 6).

#### 3.3.3. Heavy metals

**Figure 7.**
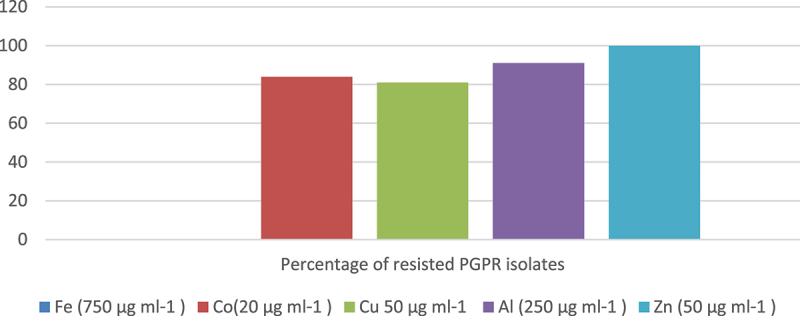
Heavy metal resistance of PGPR isolates.

The tolerance of 82 rhizobacterial isolates to varying concentrations of heavy metals was investigated in this study (Figure 7). Remarkably, all the isolates (100%) displayed resistance to zinc (50-μg ml^−1^). Additionally, a significant majority of the isolates, comprising 81%, 84%, and 91%, demonstrated growth even in the presence of copper (50 μg ml^−1^), cobalt (20 μg ml^−1^), and aluminum (250 μg ml^−1^), respectively. Interestingly, none of the isolates exhibited growth when the media were supplemented with the heavy metal iron (750-μg ml^−1^ Fe). Furthermore, the study found that a considerable proportion of the tested isolates, specifically 31%, 50%, and 12%, exhibited resistance to 60%, 80%, and 12% of the provided heavy metals,

#### 3.3.4. Drought

In order to evaluate the drought tolerance of the bacterial isolates, a screening process was conducted using nutrient broth supplemented with PEG 6000 at various water potentials, ranging from −0.05 MPa to −2.73 MPa. The purpose of this was to induce osmotic conditions and assess the isolates’ ability to withstand drought stress. Among the isolates, the criterion for selecting the most efficient ones was based on their performance at a 40% PEG concentration, which corresponded to the highest osmotic potential of −2.70 MPa. It is noteworthy that previous research studies had utilized PEG 6000 up to 25% (−0.73 MPa) to identify drought-tolerant isolates. However, in this study, the concentration was increased to 40% PEG to further challenge the isolates and identify the most resilient ones. Out of the 82 bacterial isolates tested with varying levels of PEG 6000 supplementation, only ten isolates demonstrated efficient growth up to the 40% PEG concentration based on the optical density (OD) at 600 nm (Table 2). Notably, two isolates, specifically AAURB12 and AAURB34, displayed remarkable OD values of 0.733 and 0.643, respectively, indicating their complete tolerance to drought conditions.

**Table 2.** Growth of selected bacterial isolates against moisture stress under *in-vitro* conditions.

Isolates	–0.05 Mpa	–0.65 Mpa	–1.5 Mpa	–2.17 Mpa	–2.70 Mpa
AURB6	0.762	0.561	0.277	0.129	0.082
AURB34	2.112	1.492	1.022	0.891	0.643
AURB29	0.741	0.465	0.204	0.081	0.033
AURB15	0.702	0.435	0.221	0.081	0.022
AURB20	0.963	0.821	0.541	0.221	0.099
AURB14	0.734	0.532	0.244	0.077	0.035
AURB19	1.344	1.134	0.987	0.733	0.434
AURB56	1.03	0.879	0.793	0.572	0.428
AURB25	1.344	1.117	0.991	0.723	0.497
AURB11	1.203	0.723	0.438	0.232	0.113
AURB12	2.066	1.501	1.364	1.092	0.733
AURB54	0.811	0.561	0.281	0.069	0.027
AURB2	0.982	0.741	0.533	0.122	0.072
AURB43	0.609	0.433	0.216	0.069	0.032
AURB73	0.611	0.403	0.117	0.067	0.028
AURB28	0.911	0.811	0.691	0.531	0.416
AURB49	0.955	0.688	0.366	0.091	0.052
AURB39	0.721	0.355	0.123	0.088	0.033
AURB35	1.023	0.897	0.634	0.287	0.099
AURB10	1.866	1.233	0.877	0.533	0.477

### 3.4. In vitro screening for bio-control traits

#### 3.4.1. Hydrolytic enzyme production and HCN

**Figure 8.**
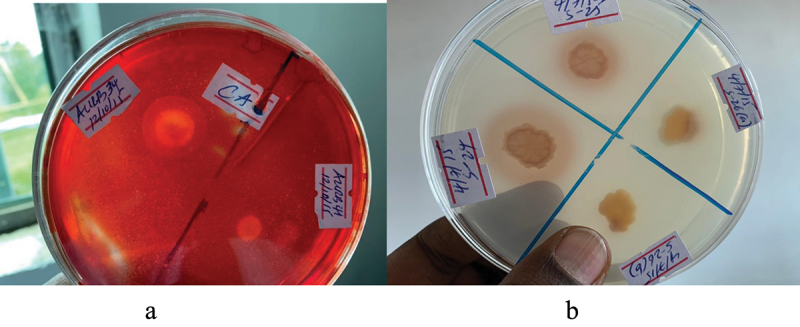
Lytic Enzyme Production of PGPR. (A): PGPR isolates exhibiting cellulose enzyme production show the formation of a halo zone when the media is flooded with Congo Red dye. The halo zone indicates the degradation of cellulose by the cellulose enzyme. (B): PGPR isolates displaying protease enzyme production exhibit a clear zone around the colony, indicating the proteolytic activity of the enzyme that leads to the breakdown of proteins in the surrounding area.

The assessment of proteolytic enzyme production, specifically protease, was carried out using skim milk agar (SMA) medium. Surprisingly, out of the 82 tested isolates, 43% (19) exhibited distinct, clear, and prominent zones of clearance around their colonies, indicating their capability to produce protease enzymes. Similarly, the cellulose synthesis capability or cellulase enzyme production was evaluated by spot inoculating all isolates on cellulose agar media. Interestingly, only 40% (17) of the isolates displayed a clear halo zone around their colonies, suggesting a positive result for cellulose synthesis (Figure 8).

**Figure 9.**
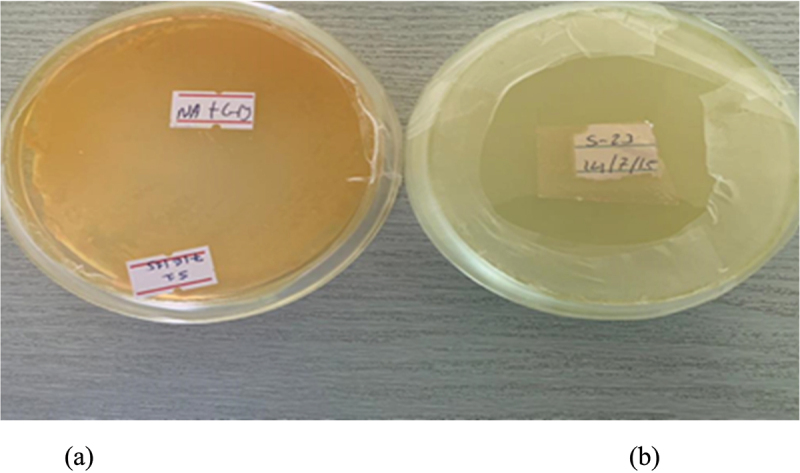
Production of Hydrogen Cyanide (HCN) by PGPR. (A) PGPR strains that produce HCN exhibit a positive reaction, indicated by the presence of HCN production. (B) PGPR strains that do not produce HCN show a negative reaction, confirming the absence of HCN production.

The HCN production ability of the 82 isolates was determined using the picric acid assay. Surprisingly, only two rhizobacterial isolates namely AAUR 12 and AAUR 34 demonstrated moderate HCN production (++), while the other 17 isolates showed weak HCN production (Figure 9). In contrast, the remaining 63 isolates did not exhibit any change in the yellow color of the picric acid solution-treated filter paper, indicating lack of HCN production.

#### 3.4.2. Intrinsic antibiotic resistance (IAR)

**Figure 10.**
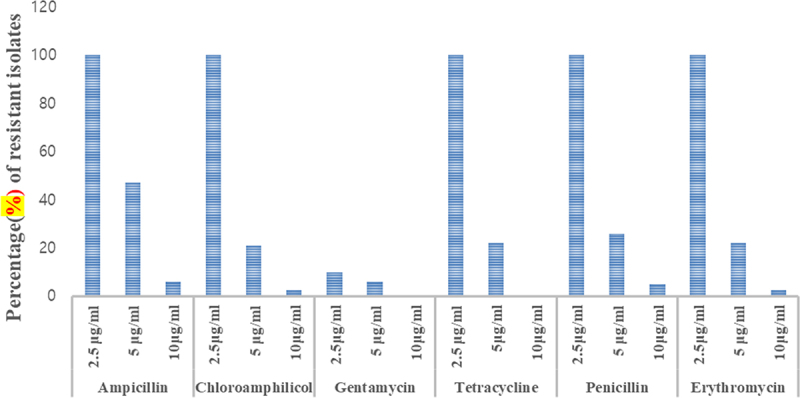
Antibiotics tolerance of PGPR isolates.

The rhizobacterial isolates exhibited variations in their IAR pattern as depicted in Figure 10. A total of 82 isolates were tested for resistance against six different antibiotics at varying concentrations of 2.5, 5, and 10 μg/mL. The results indicated that all the tested isolates displayed tolerance to the tested antibiotics at a concentration of 2 μg/mL, with the exception of gentamicin. At a concentration of 5 μg/mL, approximately half of the isolates exhibited tolerance to ampicillin compared to the other antibiotics. Notably, only a few isolates demonstrated resistance to the tested antibiotics at a concentration of 10 μg/mL, except for gentamicin and tetracycline, which showed no growth. Isolates AURB10 and AURB34 exhibited resistance to both erythromycin and chloramphenicol as well as penicillin, at a concentration of 10 μg/ml. Additionally, isolates AAURB12 and AURB19 demonstrated resistance to chloramphenicol and erythromycin, respectively, at the same concentration of 10 μg/ml.

#### 3.4.3. Antifungal activity

In this study, all the isolates were subjected to screening against the destructive fungal pathogen *Fusarium oxysporum*, which is known to negatively impact the vascular system of groundnut plants, resulting in wilting, stunting, and ultimately the death of the plant. The aim was to identify potential isolates that exhibit inhibitory effects against this fungal pathogen.

The obtained results showed that out of the 82 isolates tested, only five isolates exhibited inhibition against the targeted fungal pathogen. Notably, isolates AAUR34 and AAUR12 showed relatively stronger antagonistic effects with inhibition percentages of 38% and 46%, respectively.

**Figure 11.**
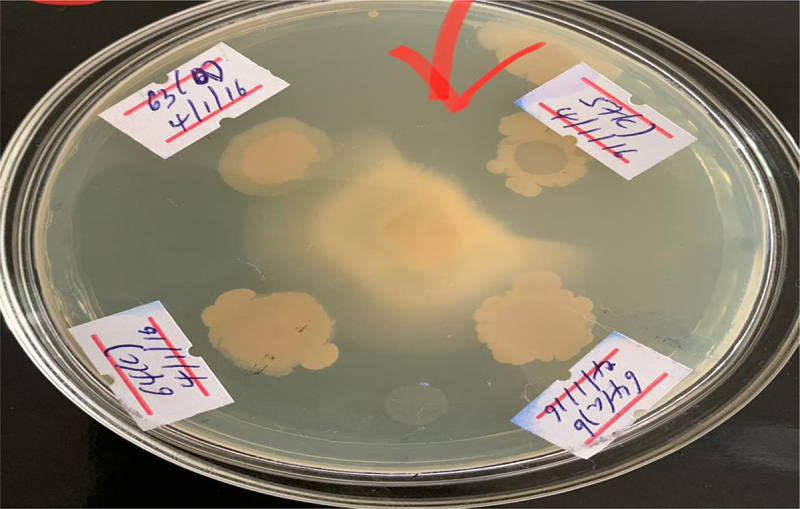
Illustrates the antifungal activity of the isolates of Plant Growth Promoting Rhizobacteria.

### 3.5. Identification of bacteria using MALDI-TOF

The utilization of Matrix-assisted laser desorption/ionization-time of flight mass spectrometry (MALDI-TOF MS) is becoming increasingly prevalent in the field of bacterial identification, particularly in the context of identifying plant growth-promoting rhizobacterial (PGPR) isolates. Among the 20 selected PGPR isolates, with multiple plant growth-promoting (PGP) characteristics, a total of 12 isolates were successfully identified at the species level, attaining a score of 2.0 or higher (Table 3). However, for the remaining isolates, identification was limited to the genus level, although the database provided suggestions for the most suitable species matches.

**Table 3.** Bacterial identification based on Matrix-assisted laser desorption/ionization-time of flight mass spectrometry.

Isolate code	MALDI TOF MS Based ID	Score
AURB6	*Enterobacter cloacae*	2.02
AURB34	*Bacillus pumilus*	2.01
AURB29	*Staphylococcus sciuri*	1.71
AURB15	*Corynebacterium accolens*	1.85
AURB20	*Staphylococcus aureus*	1.84
AURB14	*Enterobacter asburiae*	2.29
AURB19	*Staphylococcus warneri*	1.86
AURB56	*Enterobacter asburiae*	1.71
AURB25	*Staphylococcus equorum*	2.26
AURB11	*Staphylococcus xylosus*	2.15
AURB12	*Bacillus megatrium*	2.05
AURB54	*Staphylococcus xylosus*	2.1
AURB2	*Enterobacter asburiae*	2.25
AURB43	*Staphylococcus equorum*	2.29
AURB73	*Acinetobacter calcoaceticus*	1.96
AURB28	*Staphylococcus pasteuri*	2.25
AURB49	*Rhizobium leguminosarum*	2.21
AURB39	*Enterobacter aerogenes*	1.94
AURB35	*Staphylococcus equorum*	2.07
AURB10	*Enterobacter asburiae*	1.71

## 4. Discussion

Plant growth-promoting rhizospheric bacteria play a vital role in enhancing plant health and growth, especially in environments prone to drought.^27^ These bacteria are widely distributed in soil and engage in various activities that facilitate the production and secretion of substances beneficial for plant development.^28^ Through the utilization of colony morphology and gram staining techniques, this study reveals the presence of a diverse and abundant microbial community in the groundnut rhizosphere soil at the investigated site, consisting of gram-negative and gram-positive bacteria in varying proportions.

During this study, a comprehensive collection of 82 bacteria was obtained from soil samples taken from various semi-arid regions where groundnuts are cultivated (Figure 1). Among these isolates, 75 were identified as gram-negative bacteria, while the remaining seven isolates were categorized as gram-positive bacteria. Subsequently, the isolated strains underwent screening to assess their potential plant growth-promoting characteristics, stress tolerance abilities, and bio-control properties. Furthermore, the isolates were identified at the genus and species level using MALDITOF, allowing for the identification of those strains that possess multiple advantageous traits.

The bioavailable form of phosphorus in soil is typically found in very low concentrations, usually at levels of 1 ppm or less, as reported by Goldstein and Krishnaraj.^29^ Interestingly, various microorganisms have been found to possess the ability to solubilize phosphate, with those in the rhizosphere being more abundant and potent than those outside of it.^30^ In vitro testing of phosphate solubilization among the 82 tested isolates revealed that 23% exhibited a clear and yellow-colored halo zone around their colonies, indicating potent phosphate solubilization activity. The top three isolates with the highest efficiency in solubilizing phosphate were AAURB 34, AAURB 12, and AAURB 11, with p-SI values of 2.7, 2.5, and 2.4, respectively. Conversely, AAURB 02 was the least efficient, with a p-SI value of 1.75, as shown in Figure 2. Quantitatively, all selected isolates demonstrated good potential for solubilizing inorganic phosphate, as evidenced by a rapid increase in the amount of soluble P over the incubation period. Among the isolates, AURB34 (*Bacillus pumilus*) exhibited the highest potential for phosphate solubilization, with an amount of 396.48 μg P/ml, followed by AURB12 (*Bacillus megaterium*) with a phosphate solubilized amount of 240.92 μg P/ml. On the other hand, strain AURB56 (*Enterobacter asburiae*) showed the least potential, with 37.74 μg P/ml. The obtained result is in line with the discoveries of^31^ who identified the peak level of phosphorus solubilization by rhizospheric bacteria isolated from groundnut rhizosphere through in vitro testing after seven days of incubation. A significant relationship was observed between p-SI and soluble P level.

The plant hormone indole-3-acetic acid (IAA) is widely recognized as the primary endogenous auxin involved in regulating key growth processes within plant systems. Prior research has demonstrated IAA’s significant influence over root and shoot elongation through mechanisms of cell wall extension.^32^ In the present study, the majority (68%) of bacterial isolates obtained were found to produce detectable levels of IAA. Quantitative analysis revealed a broad range in IAA production levels across these auxin-synthesizing bacteria, varying from 5.5 to 84.6 micrograms per milliliter (Figure 4). These findings are consistent with the results published by Diop et al.^33^ who also observed variation in IAA production rates amongst distinct bacterial strains within their study. Specifically, Diop et al. proposed that rhizobacterial strains able to synthesize IAA at levels exceeding 13.5 micrograms per milliliter can be classified as plant growth-promoting rhizobacteria (PGPR). As the bacterial isolates in the present study included some exhibiting IAA production above this 13.5-microgram threshold, the findings provide further evidence supportive of these bacteria fulfilling a PGPR role through their auxin synthesis abilities and potential to stimulate beneficial growth responses in plant hosts.

The production of ammonium by PGPR contributes to the overall nitrogen availability in the rhizosphere, benefiting plant growth and development.^34^ All of the bacterial isolates subjected to testing demonstrated the ability to produce ammonia. These findings align closely with those of^35^ who reported that all tested isolates collected from the rhizosphere were capable of ammonia production. However, these results contradict the observations made by^31^ who found that only 40% of the tested isolates from the groundnut rhizosphere exhibited ammonia production.

Rhizospheric bacteria, which are mesophiles, thrive within an optimal temperature range of 28°C to 30°C. They exhibit poor growth at temperatures below 10°C or above 37°C.^36^ Our research revealed that all isolates exhibited robust growth at 30°C, which represents the ideal temperature for the growth of rhizospheric bacteria. Additionally, half of the isolates demonstrated tolerance to 35°C, while only two isolates were able to grow at 45°C (Figure 5). These findings align with previous reports indicating that PGPR exhibit weak growth at high temperatures, such as 45 °C^37^; .^38,39^ also reported that elevated temperatures, specifically 45°C, have an inhibitory effect on the growth response of groundnut rhizosphere bacteria. Importantly, the presence of isolates capable of tolerating high temperatures holds potential for the development of inoculants that can perform effectively under such conditions.

Salinity poses a significant problem that severely affects plant health and reduces crop productivity. Plant Growth-Promoting Rhizobacteria (PGPR) employ various pathways to alleviate salt stress in the vicinity of plants. One such mechanism involves the production of compatible solutes, such as amino acids, sugars, or their derivatives, which serve as osmolytes.^40^ These osmolytes enable organisms to survive under extreme salt conditions. All the tested isolates demonstrated growth in the presence of 0.5% NaCl, but failed to grow at higher concentrations, indicating their sensitivity to salt. Similar findings were reported by^41^ who observed that increased salt concentration can have a detrimental effect on bacterial populations, both through direct toxicity and osmotic stress. However, five PGPR isolates exhibited tolerance to salt concentrations of 3% and 4%, indicating their high salt resistance (Figure 6). This ability to thrive in high sodium chloride solutions provides these native bacterial strains with a competitive advantage in the rhizosphere, enabling them to survive and interact with host plants, particularly in salt-rich soil environments.

The presence of heavy metals in soil can have harmful effects on the microbial flora, as they can disrupt the functional groups and alter the structure of biological molecules, particularly the active sites. However, certain types of plant growth-promoting rhizobacteria (PGPR), including symbiotic nitrogen-fixing bacteria, have developed the ability to thrive in the presence of metal concentrations. This resistance may be the result of intrinsic mechanisms or induced adaptations.^42^ In the present study, all the tested PGPR isolates demonstrated a high resistance to zinc at a concentration of 50 μg mL-1. However, they exhibited a high sensitivity to iron at 750 μg mL-1. None of the isolates were able to tolerate 100% of all the provided heavy metal concentrations, which is in contrast to the research findings of^43^ where at least four isolates showed 100% tolerance to the tested heavy metals. Interestingly, some of the tested PGPR isolates were able to tolerate 80% of the provided heavy metal concentration, while only 7% of the tested isolates could tolerate 40% of the tested heavy metals.

One of the defining characteristics of Plant Growth-Promoting Rhizobacteria (PGPR) is their capacity to enhance drought resilience in plants. Based on our analysis, all tested PGPR isolates exhibited tolerance to an osmotic potential of −0.05 MPa. However, when subjected to an extreme osmotic potential of −2.7 MPa, the majority (88%) of isolates demonstrated high sensitivity. Remarkably, only 0.4% of isolates completely tolerant of such low water conditions (Table 2). These findings correlate strongly with research conducted by Nishu et al.^44^ who also observed that merely two bacterial species promoted plant growth and survived at a high Polyethylene glycol (PEG) concentration simulating 40.5% drought stress. The PGPR isolates capable of thriving in a medium with restricted water availability can therefore be considered as drought-tolerant. This tolerance appears primarily attributed to their potential to form biofilm structures and secrete extracellular polymeric substances. Such adaptive mechanisms likely enable drought-tolerant PGPR to maintain hydration and nutrient/water exchange with host plant roots, even under extreme water-deficit environments. Further studies optimizing drought-resilient PGPR formulation and application show promise for developing sustainable agriculture practices in arid regions worldwide.^43^ Such a feature is of great importance for the restoration of degraded lands under water-stressed conditions, as these potential isolates can be used for inoculation purposes.

The synthesis of hydrolytic enzymes by plant growth-promoting rhizobacteria (PGPR) is a crucial mechanism for sustainable plant disease management, as it helps combat plant pathogens. These enzymes are responsible for breaking down the cell walls of fungal pathogens, leading to their demise.^45^ PGPR strains actively produce hydrolytic enzymes such as chitinase, protease, and cellulase, which contribute to the degradation of phyto-pathogens. These antagonistic properties of hydrolytic enzymes play a significant role in biocontrol efforts.^46^ To assess the potential of bacterial antagonists in this study, phenotypic characterization was conducted in vitro to evaluate their ability to produce protease, chitinase, and cellulase. The results revealed that the production of these enzymes was the most prominent trait observed among the antagonists. Specifically, only 12% and 17% of the tested isolates demonstrated positive protease and cellulase enzyme production, respectively (Figure 8). However, none of the isolates exhibited positive chitinase activity.

PGPR strains that possess the ability to produce hydrogen cyanide (HCN) secrete hydrogen cyanide synthases, which aid in breaking down the cell walls of pathogenic microorganisms.^47^ In this study, the bacterial isolates were screened for their capacity to produce hydrogen cyanide. Out of the 82 tested PGPR isolates, only two isolates exhibited moderate production, while 17 isolates showed weak production of hydrogen cyanide (Figure 10). It is noteworthy that regardless of the host plant type, out of the 22-soybean rhizosphere isolates tested, which were obtained from Ethiopian soil, only one isolate demonstrated HCN production.^48^ Similarly, previous research by Djebaili et al.^49^ reported that a mere 3% of rhizobacterial strains, belonging to different genera and species, were capable of producing HCN, indicating the infrequent occurrence of HCN production among various rhizobacterial species. Overall, the presence, absence, and intensity of hydrogen cyanide production can play a significant role in determining the antagonistic potential of bacteria against phytopathogens.

All the isolated strains of slow-growing PGPR demonstrated tolerance to the provided antibiotics at a concentration of 2 µg/ml, and 43% of the isolates exhibited resistance to the tested antibiotics at a concentration of 5 µg/ml. Notably, certain isolates, specifically AURB10 and AURB34, displayed resistance to chloramphenicol and penicillin even at a concentration of 10 µg/ml. In a similar vein, a previous study^50^ reported that 76% of local rhizobacterial isolates from Ethiopia showed resistance to chloramphenicol. Contrasting the slow-growing groundnut isolates, the fast-growing isolates were found to be more susceptible to all tested antibiotics. This observation aligns with previous research by^51^ who characterized rhizosphere bacteria in soybean and determined that fast-growing strains tend to be more sensitive to antibiotics compared to slow-growing strains. The variation in tolerance and sensitivity among the isolates at different concentrations could contribute to a more harmonious coexistence with antibiotic-producing soil microorganisms in the soil.

To assess the effectiveness of selected PGPR isolates in antagonizing soil-borne fungal isolates, specifically F. oxysporum, a dual culture method was utilized, and the percentage of growth inhibition was recorded. The plate assay results demonstrated that only 6% of the tested PGPR isolates possessed the ability to impede the growth of the pathogenic fungi and exhibited inhibitory effects against F. oxysporum (Figure 11). Conversely, the remaining strains did not exhibit any antifungal activity. Notably, among the five strains that displayed inhibitory effects, AURB 12 and AURB 28 exhibited a higher percentage of growth suppression compared to the other tested strains. These findings are consistent with a study conducted by^52^ who isolated PGPR strains and observed that 4% of the strains effectively suppressed the growth of Fusarium spp. By introducing strains with antifungal capabilities and the potential to reduce crop loss, a significant decrease in damping-off or wilt disease in groundnut plants can be achieved. Furthermore, these biocontrol agents have been reported to produce toxic metabolites, enzymes, or volatile compounds that exert inhibitory effects on soil-borne pathogens.

The MALDI-TOF MS technique was employed to rapidly and cost-effectively identify 20 selected bacterial isolates (Table 3). This method serves as a valuable tool for identifying unknown bacterial cultures. In addition to traditional gram staining techniques, MALDI-TOF MS confirmed the categorization of isolates as either gram-negative or gram-positive. Specifically, 13 isolates were identified as gram-positive, while the remaining seven were gram-negative. Many researchers have utilized this approach to identify plant growth-promoting (PGP) bacteria associated with plants, as shown by Tani et al. in 2015. The success of MALDI-TOF MS relies on extensive and reliable reference databases. Therefore, the capabilities of MALDI-TOF MS are expected to improve as more plant-associated bacteria are added to existing databases. This will support ever more efficient identification of beneficial microbes for agricultural applications.

## 5. Conclusion

In summary, this study screened 82 soil bacteria isolated from groundnut fields in Ethiopia to identify promising plant growth-promoting and biocontrol candidates. Two isolates, designated AAURB34 and AAURB12, demonstrated consistently high performances across various tests. These isolates solubilized phosphate at the highest levels, produced elevated quantities of the plant hormones indole-3-acetic acid and ammonia, and showed robust tolerance to salt and drought stresses. Both AAURB34 and AAURB12 also generated protease and cellulase enzymes in high amounts, suggesting strong antagonistic activity against fungal pathogens. Given their multi-faceted plant growth-promoting traits, AAURB34 and AAURB12 appear to be potent candidates for developing microbial inoculants to boost groundnut yields and resilience to environmental stresses. Further greenhouse and field experiments are still needed to validate their actual plant growth-promoting effectiveness under real agricultural conditions.
